# NAVIGATING CAREWORKER CURRICULUM REVISION IN SOUTH KOREA: CHARTING COURSES FOR ELEVATING PRACTICE COMPETENCIES

**DOI:** 10.1093/geroni/igad104.3486

**Published:** 2023-12-21

**Authors:** Jihyun Park, Jinhak Kim, Mira Sung

**Affiliations:** Pyeongtaek University, Pyeongtaek, Gyeonggi, Republic of Korea; Korean Human Resource Development Institute for Health and Welfare, Osong, Chungcheong, Republic of Korea; Yong-In Arts & Science University, Yongin, Gyeonggi, Republic of Korea

## Abstract

The purpose of this study is to comprehensively investigate and analyze the process of revising the careworker curriculum in South Korea with a primary focus on augmenting practice competencies. This research involved an analysis of the existing 240-hour curriculum, complemented by a comprehensive literature review. Additionally, Focus Group Interviews were conducted with a cohort of 20 present careworkers to effectively identify the prevailing needs within the practice field. This study expanded the curriculum duration from 240 to 320 hours. Subsequently, pilot courses were executed, incorporating enhanced content related to dementia care, humanity in caregiving, and infectious disease management. These modifications were delivered via webinars to 64 active care workers, a method chosen due to the constraints imposed by the COVID-19 pandemic. This study employed a pre-post design to inquire about the perceptions of older adults and their job skills. Fifty-four female care workers (84.2%) and eleven male care workers (15.8%) participated in the study, with an average age of 52 years, experiencing 3 years and 8 months. Several areas exhibited statistical significance; understanding and managing depression and anxiety (2.175*), supporting leisure activities(2.956**), implementing abuse prevention behaviors (3.187**), creating a safe environment(2.496**), assisting with mobility(2.978**), aiding in illness management(2.805**), transferring responsibilities(2.726**), documenting cases(2.238*), and overseeing caregiver health and safety(2.805*). Based on our findings, we have devised a plan to incorporate the following content within the additional 80 hours. In a broader context, this study contributes to the ongoing discourse on caregiver education and training in South Korea.

